# Failure of functional imaging with gallium-68-DOTA-D-Phe1-Tyr3-octreotide positron emission tomography to localize the site of ectopic adrenocorticotropic hormone secretion: a case report

**DOI:** 10.1186/1752-1947-5-405

**Published:** 2011-08-23

**Authors:** Linsey U Gani, Emily J Gianatti, Ada S Cheung, George Jerums, Richard J MacIsaac

**Affiliations:** 1 Endocrine Centre and Department of Medicine, Austin Health and University of Melbourne, PO BOX 5444, Heidelberg West 3081, Victoria, Australia; 2 Department of Endocrinology and Diabetes, St Vincent's Hospital and University of Melbourne, PO BOX 2900 Fitzroy 3065, Victoria, Australia

## Abstract

**Introduction:**

The diagnostic efficacy of biochemical and imaging modalities for investigating the causes of Cushing's syndrome are limited. We report a case demonstrating the limitations of these modalities, especially the inability of functional imaging to help localize the site of ectopic adrenocorticotropic hormone secretion.

**Case presentation:**

A 37-year-old Arabian woman presented with 12 months of progressive Cushing's syndrome-like symptoms. Biochemical evaluation confirmed adrenocorticotropic hormone -dependent Cushing's syndrome. However, the anatomical site of her excess adrenocorticotropic hormone secretion was not clearly delineated by further investigations. Magnetic resonance imaging of our patient's pituitary gland failed to demonstrate the presence of an adenoma. Spiral computed tomography of her chest only revealed the presence of a non-specific 7 mm lesion in her left inferobasal lung segment. Functional imaging, including a positron emission tomography scan using 18-fluorodeoxyglucose and gallium-68-DOTA-D-Phe1-Tyr3-octreotide, also failed to show increased metabolic activity in the lung lesion or in her pituitary gland. Our patient was commenced on medical treatment with ketoconazole and metyrapone to control the clinical features associated with her excess cortisol secretion. Despite initial normalization of her urinary free cortisol excretion rate, levels began to rise eight months after commencement of medical treatment. Repeated imaging of her pituitary gland, chest and pelvis again failed to clearly localize a source of her excess adrenocorticotropic hormone secretion. The bronchial nodule was stable in size on serial imaging and repeatedly reported as having a nonspecific appearance of a small granuloma or lymph node. We re-explored the treatment options and endorsed our patient's favored choice of resection of the bronchial nodule, especially given that her symptoms of cortisol excess were difficult to control and refractory. Subsequently, our patient had the bronchial nodule resected. The histological appearance of the lesion was consistent with that of a carcinoid tumor and immunohistochemical analysis revealed that the tumor stained strongly positive for adrenocorticotropic hormone. Furthermore, removal of the lung lesion resulted in a normalization of our patient's 24-hour urinary free cortisol excretion rate and resolution of her symptoms and signs of hypercortisolemia.

**Conclusion:**

This case report demonstrates the complexities and challenges in diagnosing the causes of adrenocorticotropic hormone -dependent Cushing's syndrome. Functional imaging may not always localize the site of ectopic adrenocorticotropic hormone secretion.

## Introduction

The diagnostic efficacy of biochemical and imaging modalities for localizing the anatomical site of ectopic adrenocorticotropic hormone (ACTH) secretion are limited. Somatostatin receptor scintigraphy (SRS), using the ligand 111-indium-pentetreotide, has traditionally been the functional imaging technique used, but it's usefulness has been questioned [1]. Recently, positron emission tomography (PET) scanning using gallium-68-DOTA-D-Phe1-Tyr3-octreotide (DOTATOC) has been reported to be a superior modality for detecting neuroendocrine tumors [2]. However, here we describe a case where this functional imaging technique failed to localize the site of ectopic ACTH secretion.

## Case presentation

A 37-year-old Arabian woman was referred to our endocrinology clinic with 12 months of progressive weight gain of 30 kg, hirsutism, acne, alopecia, lethargy, amenorrhea and marked anxiety. An examination revealed features of Cushing's syndrome with rounded face, buffalo hump, abdominal striae and proximal muscle weakness. Investigations confirmed excess cortisol production. Her 24-hour urinary free cortisol excretion was 1870 nmol/day (normal range 40-450 nmol/day), her midnight salivary cortisol level was 121 nmol/L (normal range < 9 nmol/L) and after a 1 mg overnight dexamethasone suppression test her serum cortisol level was 597 nmol/L (expected value < 50 nmol/L). Her serum ACTH level was elevated at 55.8 and 55.1 ng/L on two separate occasions (normal range 7-63.2 ng/L), consistent with a diagnosis of ACTH-dependent Cushing's syndrome. Magnetic resonance imaging (MRI) of her pituitary gland did not reveal the presence of an adenoma.

**Figure 1:**
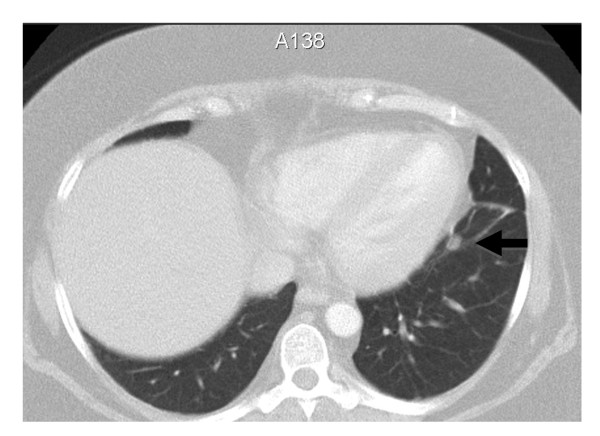
**Transverse image of a chest CT scan showing a small 7 mm inferobasal segment lesion (arrow) in the left lower lobe**.

Localizing the source of excess ACTH secretion was challenging. Inferior petrosal sinus (IPS) sampling was difficult due to a left petrosal sinus anatomical variation. However, it demonstrated a central to peripheral ACTH gradient of less than three, consistent with ectopic ACTH secretion. This diagnosis was supported by failure of cortisol suppression (472 nmol/L) after an 8 mg overnight dexamethasone suppression test. Computed tomography (CT) of her chest, abdomen and pelvis only revealed a well circumscribed 7 mm left inferior basal lung segment lesion. This was reported to most likely represent a benign granuloma or a small lymph node (Figure 1).

Due to the wide range in sensitivity and specificity of the high dose dexamethasone suppression test (59-92% and 67-100%, respectively) and the inability to successfully catheterize her left IPS, further dynamic biochemical tests were performed [3]. A five-hour intravenous dexamethasone test suppressed her serum cortisol level at five hours to less than 70% of basal values and a peripheral corticotrophin-releasing hormone (CRH) test showed a 58% increase in ACTH levels from baseline. Contrary to preceding results, these findings could be interpreted to suggest the presence of a pituitary source for her excess ACTH secretion. However, PET scanning using 18-fluorodeoxyglucose (FDG) and gallium-68-DOTATOC failed to show increased metabolic activity in the lung lesion or in her pituitary gland.

Serial CT scanning of her chest, abdomen and pelvis over 18 months failed to definitively localize a source of ectopic ACTH production. The well circumscribed 7 mm left inferior basal lung segment lesion was reported as stable in size over this time. A repeat MRI of her pituitary gland once again did not reveal the presence of an adenoma.

**Figure 2:**
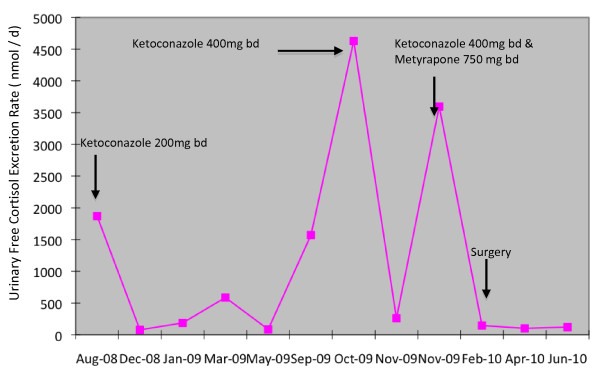
**Pattern of 24-hour urinary free cortisol secretion (normal range: 25-360 nmol/day) in response to various treatment modalities**. Note the horizontal axis is not to scale.

Given the failure of biochemical or imaging techniques to localize the site of excess ACTH secretion, medical therapy was initiated with ketoconazole. However, combination treatment with metyrapone was required after eight months due to rising 24-hour urinary free cortisol levels and progressive symptoms of weight gain, lethargy, depression and anxiety. Despite combination medical therapy there was still a progressive rise in 24-hour urinary free cortisol levels (Figure 2). As a result, our patient again developed florid symptoms of weakness, depression and anxiety which limited her daily activities and interpersonal relationships.

Given the failure of medical therapy to control her symptoms, other potential treatment options were discussed with our patient. These included progressing to bilateral adrenalectomy or resection of the lung lesion, which was the only possible anatomical site of ectopic ACTH secretion located so far. Unfortunately, the lung lesion was reported to be lying adjacent to the pericardium which negated a minimally invasive surgical approach to remove it. Despite this our patient still strongly favored proceeding to resection of the lung lesion even though this would require an open procedure. Hence, an open thoracotomy to remove the lung lesion was performed 18 months after her initial presentation.

Histological examination of the resected nodule showed a 9 mm well circumscribed tumor surrounding a bronchus, with features consistent with a carcinoid tumor. Immunohistochemical analysis revealed that the tumor stained strongly positive for chromogranin, synaptophysin and ACTH. Postoperative recovery was uneventful and perioperative corticosteroid replacement was progressively weaned. Clinically, her symptoms of Cushing's syndrome slowly abated. She achieved a normal 24-hour urinary free cortisol excretion off all treatment four months after surgery. Our patient remains well with no clinical or biochemical evidence of cortisol excess seven months after her surgery.

## Discussion

This case illustrates the difficulty in diagnosing a pituitary or an ectopic source of ACTH-dependent Cushing's syndrome. A wide variability in the sensitivity and specificity of current biochemical dynamic tests has been reported (Table 1). None of the current diagnostic tests are able to differentiate between pituitary and ectopic ACTH syndrome with 100% sensitivity and specificity. Thus there is a need for a combination of tests to help determine the cause of Cushing's syndrome.

**Table 1:** Reported sensitivity and specificity of commonly utilized dynamic biochemical diagnostic tests for determining the site of excess ACTH secretion

Diagnostic Test	Sensitivity	Specificity
Overnight high dose dexamethasone suppression test (8 mg) [3]	59-92%	67-100%

IV dexamethasone suppression test [5]	95-100%	40-90%

Ovine CRH stimulation test [5]	85-93%	85-90%

IPS sampling (central: peripheral gradient) [6]	81-85%	90-100%

Furthermore, functional imaging may not always assist in localizing an anatomical site of excess ACTH secretion (Table 2). In some instances, ectopic ACTH-secreting tumors can be detected by SRS using 111-indium-pentetreotide, or as highlighted in a recent case reported in this journal, with technetium-99 m-labelled octreotide acetate [4]. However, the sensitivity of SRS for detecting occult tumors that secrete ACTH only ranges from 30 to 53% [1]. In contrast, some preliminary reports have suggested that PET scanning using gallium-68-DOTATOC yields a higher detection rate of neuroendocrine tumors compared to SRS [2]. Despite this, the limitations of even this technique to localize an ectopic source of ACTH secretion are highlighted by this case.

**Table 2:** Reported sensitivity of current imaging modalities for localizing the site of ectopic ACTH secretion

Imaging Modalities	Sensitivity	Specificity
CT and MRI [7]	53 -74%	n/a

111-indium pentetreotide SRS [1]	33-88%	n/a

18-FDG PET scanning [8]	35-66%	n/a

Gallium 68-DOTATOC PET scanning [2]	82%	n/a

When all modalities fail to localize a source of ectopic ACTH, the role of clinical judgment plays a significant role. Ongoing monitoring of the patient, combined with a relevant discussion of risks and benefits of different therapeutic options led to a decision to proceed to removal of the small bronchial nodule. This nodule was subsequently confirmed to be an ACTH-secreting carcinoid tumor. Embarking on this decision despite there being no definitive preoperative confirmation that the nodule was the source of her ectopic ACTH production resulted in a cure of our patient's Cushing's syndrome.

## Conclusion

We have shown the limitations of the currently available diagnostic tools in differentiating pituitary or ectopic sources of ACTH-dependent Cushing's syndrome. Furthermore, despite significant advances in radiological and nuclear medicine imaging modalities, the localization of the site of ectopic ACTH may still not be possible.

## Consent

Written informed consent was obtained from the patient for publication of this case report and any accompanying images. A copy of the written consent is available for review by the Editor-in-Chief of this journal.

## Competing interests

The authors declare that they have no competing interests.

## Authors' contributions

EG, AC and RM analyzed and interpreted the patient's data and were involved in the patient's care. LG was a major contributor to writing the manuscript. All authors read and approved the final manuscript.
